# Socioeconomic status and diabetes technology use in youth with type 1 diabetes: a comparison of two funding models

**DOI:** 10.3389/fendo.2023.1178958

**Published:** 2023-08-21

**Authors:** Kate E. Lomax, Craig E. Taplin, Mary B. Abraham, Grant J. Smith, Aveni Haynes, Ella Zomer, Katrina L. Ellis, Helen Clapin, Sophia Zoungas, Alicia J. Jenkins, Jenny Harrington, Martin I. de Bock, Timothy W. Jones, Elizabeth A. Davis

**Affiliations:** ^1^ Department of Endocrinology and Diabetes, Perth Children’s Hospital, Nedlands, WA, Australia; ^2^ Children’s Diabetes Centre, Telethon Kids Institute, The University of Western Australia, Perth, WA, Australia; ^3^ Centre for Child Health Research, The University of Western Australia, Perth, WA, Australia; ^4^ Division of Paediatrics within the Medical School, The University of Western Australia, Perth, WA, Australia; ^5^ School of Public Health and Preventive Medicine, Monash University, Melbourne, VIC, Australia; ^6^ Diabetes and Vascular Medicine, Baker Heart and Diabetes Institute, Melbourne, VIC, Australia; ^7^ NHMRC Clinical Trials Centre, The University of Sydney, Sydney, NSW, Australia; ^8^ Division of Endocrinology, Women’s and Children’s Health Network, North Adelaide, SA, Australia; ^9^ Faculty of Health and Medical Sciences, The University of Adelaide, Adelaide, SA, Australia; ^10^ Department of Paediatrics, University of Otago, Christchurch, New Zealand

**Keywords:** type 1 diabetes (T1D), paediatrics, socioeconomics, equity, model of care, technology

## Abstract

**Background:**

Technology use, including continuous glucose monitoring (CGM) and insulin pump therapy, is associated with improved outcomes in youth with type 1 diabetes (T1D). In 2017 CGM was universally funded for youth with T1D in Australia. In contrast, pump access is primarily accessed through private health insurance, self-funding or philanthropy. The study aim was to investigate the use of diabetes technology across different socioeconomic groups in Australian youth with T1D, in the setting of two contrasting funding models.

**Methods:**

A cross-sectional evaluation of 4957 youth with T1D aged <18 years in the national registry was performed to determine technology use. The Index of Relative Socio-Economic Disadvantage (IRSD) derived from Australian census data is an area-based measure of socioeconomic status (SES). Lower quintiles represent greater disadvantage. IRSD based on most recent postcode of residence was used as a marker of SES. A multivariable generalised linear model adjusting for age, diabetes duration, sex, remoteness classification, and location within Australia was used to determine the association between SES and device use.

**Results:**

CGM use was lower in IRSD quintile 1 in comparison to quintiles 2 to 5 (p<0.001) where uptake across the quintiles was similar. A higher percentage of pump use was observed in the least disadvantaged IRSD quintiles. Compared to the most disadvantaged quintile 1, pump use progressively increased by 16% (95% CI: 4% to 31%) in quintile 2, 19% (6% to 33%) in quintile 3, 35% (21% to 50%) in quintile 4 and 51% (36% to 67%) in the least disadvantaged quintile 5.

**Conclusion:**

In this large national dataset, use of diabetes technologies was found to differ across socioeconomic groups. For nationally subsidised CGM, use was similar across socioeconomic groups with the exception of the most disadvantaged quintile, an important finding requiring further investigation into barriers to CGM use within a nationally subsidised model. User pays funding models for pump therapy result in lower use with socioeconomic disadvantage, highlighting inequities in this funding approach. For the full benefits of diabetes technology to be realised, equitable access to pump therapy needs to be a health policy priority.

## Introduction

Advanced diabetes technologies including the use of pump therapy and continuous glucose monitoring (CGM) are associated with improved glycaemic outcomes in youth with type 1 diabetes (T1D) (1–5). Sustained long-term improvements in glycaemic control and reduction of long-term diabetes associated complications including microvascular complications and cardiovascular mortality, have been demonstrated for youth and adults with T1D on pump therapy in comparison to multiple daily injections (MDI) (6–10). Current consensus guidelines recommend that youth with T1D should be offered the most advanced diabetes technologies that are affordable and available to them, with choice of device based on specific needs to promote personalised diabetes care (11, 12). Accessibility of these devices varies globally; reimbursement models differ and barriers to access persist in many populations (13, 14). A recent review of 29 European countries revealed discrepancies in CGM and pump therapy; overall, CGM was at least partially subsidised in 17 of 29 countries, whereas pump therapy was readily available in 20 of 29 countries, with other countries reporting access and reimbursement issues (15).

Furthermore, lower socioeconomic status (SES) is associated with adverse glycaemic outcomes in youth with T1D (16–19), which may be compounded or partially driven by the association of SES and use of diabetes technologies (18, 20, 21). Given the association between use of diabetes technologies and improved glycaemic outcomes, disparity in access to technology may potentiate disadvantage in low socioeconomic groups.

The association between SES and technology use in youth with T1D has not been previously investigated in the Australian population. The public hospital network and the universal health insurance system provide free or low-cost health care to all Australian citizens (22). However, Australia has two separate models for funding of advanced diabetes technologies. In April 2017, the Australian Government committed to fully subsidising CGM for children and young adults (aged <21 years) living with T1D, dramatically increasing CGM uptake (23). Insulin pump therapy is not publicly subsidised, rather pumps are accessed primarily via private health insurance or self-funding, both of which carry significant financial burden for families. Across the Australian states and territories, there is limited access to compassionate programs for low-income families to access pumps (24) and strict criteria are in place, potentially excluding many from accessing reimbursement via these pathways who would find self-funding a pump financially burdensome or impossible. Furthermore, pump provision beyond young adulthood is not usually available. These disparities in pump access are now of critical importance with the availability of new generation hybrid closed loop (HCL) systems that require insulin pump therapy combined with CGM use, and which have been shown to improve outcomes for people living with T1D (25–27).

These two distinct models for pump therapy and CGM provide the platform to investigate the impact of differing funding models on technology use in Australian youth with T1D. The aim of this study was to evaluate the association between SES and use of diabetes technologies. We hypothesized that socioeconomic disadvantage would be associated with lower use of diabetes technologies.

## Methods

### Study design and population

This is a cross-sectional population-based study of children aged <18 years with T1D receiving care at paediatric diabetes centres in Australia.

Demographic and clinical information was extracted from the Australasian Diabetes Data Network (ADDN), a prospective, longitudinal database with contributing centres comprising major diabetes clinics across Australia and New Zealand (28). Initiated in 2012, the registry has expanded to include data from 23 paediatric centres, 13 of which are in Australia. The majority of Australian children, in both urban and regional settings, receive care in public paediatric services, of which a majority share data with ADDN. Those who have not consented to share data with ADDN, and those undergoing care in the private system are not represented in this cohort’s analysis.

Data from the 1^st^ of July 2020 to the 1^st^ July 2022 were included in the study. 5913 individuals were aged <18 years and contributing data to ADDN on 1^st^ July 2022. Information recorded at the visit closest to this reference date was used to determine device use and postcode. Where information on device use was missing from this record, device use status at the previous visit was carried forward from a maximum of 2 years prior (median time difference between visit date and device use status = 0.29 years, interquartile range = 0.12 to 0.67 years). CGM and pump use were classified as binary measures (Yes/No) based on whether the device was being used at this visit. Those with missing postcode data (n=145), or who did not have a clinic visit (n=811) were excluded from the analysis, resulting in a dataset from 4957 individuals, which represents approximately 30-40% of youth living with T1D in Australia, with 14,919 individuals aged 0-20 years registered with the National Diabetes Services Scheme as of 30^th^ June 2022 (29), noting difference in age range for this reference and those included in the study.

Consent for data sharing with ADDN is obtained from individuals from their diabetes centre, usually at time of diagnosis.

### Area-based measures

*S*ocio-Economic Indexes for Areas (SEIFA) is a tool developed by the Australian Bureau of Statistics, utilising Australian census data to capture aspects of relative socioeconomic advantage and disadvantage (30). Statistical areas can then be ranked regarding SES utilising one of four specific indices. The SEIFA Index of Relative Socio-Economic Disadvantage (IRSD) was implemented in this analysis to provide an area-based indication of disadvantage, as individual-level measures of SES are not collected through ADDN. The IRSD includes 16 variables incorporating income, highest level of education, employment type and status, spoken English ability, family structure, disability needs, housing factors, internet and personal vehicle access (30). Utilisation of socioeconomic deprivation indices has been validated in other studies when individual-level measures of SES have not been prospectively collected (18, 31–33). Most recent primary residential postcode was used to determine an individual’s IRSD quintile.

In addition, the Remoteness Areas Structure within the Australian Statistical Geography Standard was used to classify, based on residential postcode, an individual’s location within Australia as being urban (major city), inner and outer regional, remote or very remote (34).

### Statistical analysis

Standard descriptive statistics were used to summarise demographic and clinical characteristics of the study population; medians and interquartile ranges for continuous measures, counts, proportions and percentages for categorical measures.

Pump and CGM use in each IRSD quintile were summarised as percentages (with 95% Wilson score confidence intervals) and chi-square tests conducted. Multivariable generalised linear models (Poisson family with log link and robust sandwich errors) were used to determine the association between SES and device use following adjustment for relevant confounders. These models included terms for IRSD quintile, remoteness classification, age, diabetes duration, sex, and individual’s location within Australia. The overall significance of IRSD quintile in predicting device use was assessed using a chi-square test of the difference in residual deviance when IRSD quintile was added to the model. Adjusted risk ratios with 95% confidence intervals, were calculated for pairwise comparisons of IRSD quintiles 2 to 5 to quintile 1.

Sensitivity models including and excluding Remoteness Area Structure were conducted to explore any model bias due to potential over-adjustment when determining the relationship between SES and device use.

## Results

Data for 4957 Australian youth living with T1D with a mean (SD) age of 12.3 (3.7) years and mean (SD) duration of diabetes of 4.8 (3.7) years were analysed (**Table 1**). Mean (SD) HbA1c for all individuals was 8.1% (1.6%)/65 (17.5) mmol/mol, reflecting previous analyses of the ADDN cohort (35). The distribution of individuals varied across the quintiles, with the majority living in postcodes associated with IRSD quintiles 4 and 5 (49.8%), the areas associated with lower relative socioeconomic disadvantage (**Table 1**).

**Table 1:** Cohort demographics.

	Study population
Total n	4957
Male (n, %)	2513 (50.7%)
Age (years)*	12.3 (3.7)
Age at diagnosis (years)*	7.5 (3.9)
Duration of diabetes (years)*	4.8 (3.7)
HbA1c %*^1^ (mmol/mol)*	8.1 (1.6) (65 (17.5))
IRSD Quintile 1 (n, %)	679 (13.7%)
IRSD Quintile 2 (n, %)	733 (14.8%)
IRSD Quintile 3 (n, %)	1080 (21.8%)
IRSD Quintile 4 (n, %)	1143 (23.1%)
IRSD Quintile 5 (n, %)	1322 (26.7%)

*Values expressed in Mean (± SD).

^1^ Missing for 515.

**Figure 1:**
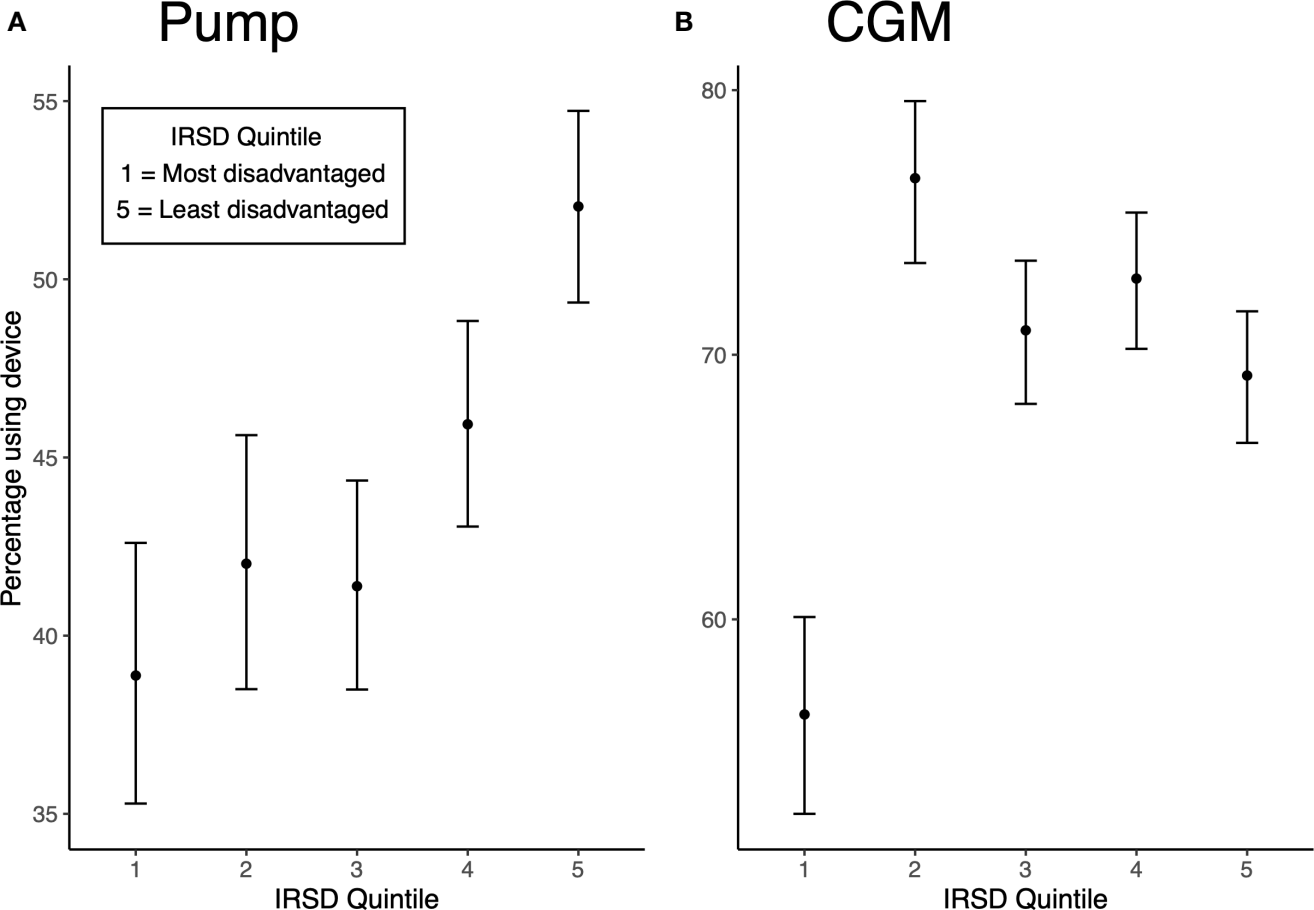
**(A)** Unadjusted percentage of pump use by IRSD quintile (with Wilson score CI’s). **(B)** Unadjusted percentage of CGM use by IRSD quintile (with Wilson score CI’s).

Pump use differed significantly by IRSD quintile (*χ* ^2^ (4, 4957) = 80.17, p<0.001). **Figure 1A** provides the percentages [with 95% Wilson score confidence intervals (CI’s)] of individuals using pumps across IRSD quintiles. Increased pump use was associated with increasing IRSD quintile, with the lowest use in the most disadvantaged quintile (38.9%) and highest use in the least disadvantaged quintile (52%). The pattern of increasing pump use with higher IRSD quintile was still observed after adjusting for age, duration of diabetes, sex, remoteness and individual’s location within Australia (see **Table 2** for adjusted risk ratios (RR_adj_) with CI’s). Compared to the most disadvantaged IRSD quintile 1, pump use was 16% more likely in quintile 2 (RR_adj_ = 1.16 [95% CI: 1.04, 1.31]), 19% more likely in quintile 3 (RR_adj_ = 1.19 [1.06, 1.33]), 35% more likely in quintile 4 (RR_adj_ = 1.35 [1.21, 1.50], and 51% more likely in the least disadvantaged quintile 5 (RR_adj_ = 1.51[1.36, 1.67]).

**Table 2:** Adjusted risk ratios (with 95% CI) of increasing use of technology use across quintiles.

IRSD Quintile	Pump use Relative Risk [95% CI]	P-value**	CGM use Relative Risk [95% CI]	P-value**
**1**	1.00	<0.001	1.00	<0.001
**2**	1.16 [1.04, 1.31]		1.35 [1.26, 1.44]	
**3**	1.19 [1.06, 1.33]		1.28 [1.19, 1.37]	
**4**	1.35 [1.21, 1.50]		1.35 [1.26, 1.45]	
**5**	1.51 [1.36, 1.67]		1.34 [1.25, 1.44]	

*The model adjusted for age, sex, duration of diabetes, remoteness and location within Australia.

**P-value of overall significance of IRSD Quintile based on a chi-square test of change in residual deviance.

CGM use also differed significantly by IRSD quintile (*χ* ^2^ (4, 4957) = 45.48, p<0.001). **Figure 1B** displays percentages (with 95% confidence intervals) of CGM use across IRSD quintiles. The lowest use was seen in quintile 1 at 56.4%, with higher but similar use across quintiles 2 to 5 (76.7% in quintile 2, 70.9% in quintile 3, 72.9% in quintile 4 and 69.2% in quintile 5). After adjusting for available sociodemographic and clinical characteristics, IRSD quintile 1 remained a predictor of less CGM use (see **Table 2** for adjusted risk ratios). Compared to the most disadvantaged quintile 1, CGM use was 35% more likely in quintile 2 (RR_adj_ = 1.35 [1.26, 1.44]), 28% more likely in quintile 3 (RR_adj_ = 1.28 [1.19, 1.37]), 35% more likely in quintile 4 (RR_adj_ = 1.35 [1.26, 1.45]), and 34% more likely in the least disadvantaged quintile 5 (RR_adj_ = 1.34 [1.25, 1.44]).

Model results were similar with or without inclusion of Remoteness Area Structure (see **Supplementary Material**).

## Discussion

This large, cross-sectional national study demonstrated an association between socioeconomic deprivation and the use of advanced diabetes technologies in youth with T1D in Australia. The differential funding models for advanced diabetes technologies in Australia provided the opportunity to explore the association between SES and technology use, which has not previously been investigated in the Australian context.

This study demonstrated that pump therapy use was higher in the least socioeconomically deprived quintiles. The pattern of pump use across quintiles is more likely to represent the ability of families living in higher quintiles to afford private health insurance or self-funding of pumps, potentially reflecting the inadequacy of the current funding model for pump therapy in Australia. The finding of increased pump use in higher quintiles may also represent other social determinants of health in higher quintiles, such as higher levels of parental education increasing understanding and acceptability of pump therapy, reduced financial barriers impacting access to health care or better English language skills, given that delivery of pump therapy education is primarily in English.

Conversely, CGM use was lowest in the most disadvantaged quintile despite fully subsidised access to this technology for all Australian youth. Similar use across quintiles 2 to 5 was observed. In 2017, the Australian government subsidised CGM use for Australian youth living with T1D. CGM uptake increased from less than 5% to 79% two years post-subsidy of CGM (23). The important but unexplained finding in our study of significantly lower CGM use in quintile 1 compared to other quintiles, in the context of a population with overall high CGM use given national subsidy, warrants further investigation. Delineating the specific barriers to accessing CGM technology in the most disadvantaged quintile has the potential to inform policy change and impact models of care in T1D. We hypothesise that factors including clinician bias in technology initiation, access to relevant mobile devices, technology acceptability and understanding which could be impacted by English language proficiency or parental level of education, could be driving this finding in the Australian population. Prospective quantitative analysis of socioeconomic factors influencing CGM use in this group compared to other quintiles, specifically investigating the impact of ethnicity, parental level of education, technology understanding, access and burden, and other potential factors, will be essential to explain the findings. Longitudinal studies rather than cross-sectional analysis will also aid in our understanding of this issue. Qualitative investigation of groups with lower technology uptake will help identify the barriers limiting the uptake of CGM in a fully subsidised funding model. This will inform Australian care providers in regard to addressing these factors as well as assist other countries with their technology funding models and implementation strategies.

IRSD quintiles 4 and 5 represent approximately half of the study cohort (combined 49.8%). Higher socioeconomic status has previously been reported to be an independent risk factor for T1D in Australia, potentially explaining this finding (36). As discussed in detail below, SEIFA statistical areas do not precisely align with Australian postcodes, and it is possible that IRSD quintile based on postcode rather than SEIFA statistical area skews a portion of the population into higher IRSD quintiles. Despite the relatively large sample size, results could also reflect a non-representative sample of the T1D paediatric population within ADDN given 4957 individuals represent approximately 30-40% of youth living with T1D in Australia as previously mentioned, and that individuals lacking postcode data or visit history were unable to be included in the analysis.

This is the first analysis of diabetes technology use in relation to SES in the Australian paediatric context. A key strength of the study design is that a large number of individuals with T1D were able to be included in the analysis. Given Australia's unique global position with differential diabetes technology funding models, the results of this study contribute evidence that further advocacy efforts for improved pump therapy access are required. The results have the potential to support advocacy and inform Australian healthcare policy in regard to technology access. This study highlights the importance of investigating if this association also exists in the Australian adult population, as funding models for diabetes technologies evolve in this cohort.

There are several limitations to this study. SEIFA IRSD quintiles represent the area-based deprivation in which an individual lives and provide an indication of socioeconomic deprivation only. Furthermore, SEIFA statistical areas do not precisely align with Australian postcode areas, which can have a wide variation of socioeconomic deprivation within them. IRSD based on an individual’s postcode should be interpreted with care. Collection of the individual-level factors influencing the SES of youth with T1D would be required to further delineate specific barriers in accessing technology. These factors include but are not limited to; parental occupation, income and level of education, ethnicity, private health insurance status, family structure and supports, however this type of data are not collected through ADDN.

The use of advanced diabetes technologies has been demonstrated to improve glycaemic outcomes in T1D (1, 2, 5), however there are barriers in accessing these technologies. Various studies have identified specific barriers including funding models (13, 37) and individual-level factors of socioeconomic deprivation including ethnicity, access to private health insurance, higher parental level of income and education (38–41). Clinician selection bias when selecting individuals to initiate diabetes technologies has also been demonstrated (42, 43) and acceptability of technology to individuals and their families, including in decision making regarding technology use, is also an important barrier to consider in the context of our findings. As glycaemic control is further impacted by socioeconomic status (19, 44, 45), non-inclusive funding models may potentiate disparities in glycaemic outcomes for youth with T1D. Considering the improvement in glycaemic control and reduction in diabetes related complications demonstrated with pump therapy and CGM, addressing barriers and improving funding models are critical goals in creating equitable access to diabetes technology in Australia.

This cross-sectional analysis of national data shows that despite universal subsidy, those in the most disadvantaged quintile utilised CGM less than those in other quintiles, where use was similar. This indicates that while subsidy is an essential first step, it is insufficient to prevent socioeconomic-based inequities in technology use. Conversely, for pump therapy, where a user pays model operates, an increase in pump use was associated with decreasing socioeconomic disadvantage. A health system that supports all youth living with T1D to access and use diabetes technologies is likely to result in improved glycaemic outcomes, with the consequent cost benefit of reduced complications, improved mental health and increased productivity. Future models of care should evolve to address barriers to accessing diabetes technology, particularly with the rapidly accumulating evidence from both research and real-world settings that advanced HCL systems improve glucose control for youth with T1D.

## Data availability statement

The raw data supporting the conclusions of this article will be made available by the authors, without undue reservation.

## Ethics statement

The studies were conducted in accordance with the local legislation and institutional requirements. All centres contributing data had Human Research or Health and Disability Ethics Committee approval for participation in ADDN. Patients provided written informed consent for data upload to ADDN. The current analysis was approved by Monash University Human Research Ethics Committee (MUHREC), Project ID 38039.

## Author contributions

All authors contributed to the development of this project and editing of the manuscript as well as the Australasian Diabetes Data Network (ADDN) study group.

## Group members of Australasian Diabetes Data Network (ADDN)

The authors drafted this manuscript on behalf of the ADDN Study Group members (18th June 2023; authors have been removed from the list): Professor Maria Craig (The Children’s Hospital at Westmead, Sydney), Dr Kym Anderson (University Hospital Geelong, Geelong), Associate Professor Sof Andrikopoulos (Australian Diabetes Society), Clinical Professor Geoff Ambler (The Children’s Hospital at Westmead, Sydney), Dr Helen Barrett (The Mater Private Hospital, Brisbane), Professor Jenny Batch (Queensland Children’s Hospital, Brisbane), Associate Professor Philip Bergman (Monash Children’s Hospital, Melbourne), Professor Fergus Cameron (Royal Children’s Hospital, Melbourne), Professor Peter Colman (The Royal Melbourne Hospital, Melbourne), Associate Professor Louise Conwell (Queensland Children’s Hospital, Brisbane), Associate Professor Andrew Cotterill (Queensland Children’s Hospital, Brisbane), Dr Chris Cooper (University Hospital Geelong, Geelong), Professor Jennifer Couper (Women’s and Children’s Hospital, Adelaide), Professor Kim Donaghue (The Children’s Hospital at Westmead, Sydney), Dr Jan Fairchild (Women’s and Children’s Hospital, Adelaide), Dr Gerry Fegan (Fiona Stanley Hospital, Perth), Dr Spiros Fourlanos (The Royal Melbourne Hospital, Melbourne), Associate Professor Sarah Glastras (Royal North Shore Hospital, Sydney), Dr Leonie Gray (Mater Medical Centre, Rockhampton), Associate Professor Shane Hamblin (Western Health, Melbourne), Professor Paul Hofman (University of Auckland, New Zealand), Associate Professor Dianne Jane Holmes-Walker (Westmead Hospital, Sydney), Dr Neville Howard (The Children’s Hospital at Westmead, Sydney), Dr Michelle Jack (Royal North Shore Hospital, Sydney), Dr Steven James (University of the Sunshine Coast, Caboolture), Dr Craig Jefferies (Starship Children’s Health, New Zealand), Dr Stephanie Johnson (Queensland Children’s Hospital, Brisbane), Dr Jeff Kao (Royal Children’s Hospital, Melbourne), Associate Professor Bruce R King (John Hunter Children’s Hospital, Newcastle), Dr Antony Lafferty (The Canberra Hospital, Canberra), Ms Michelle Martin (Illawarra Shoalhaven Diabetes Service, Wollongong), Dr Robert McCrossin (Gladstone Hospital, Gladstone), Dr Mark Pascoe (Royal Hobart Hospital, Hobart), Dr Ryan Paul (Waikato Hospital, New Zealand), Dr Alexia Peña (The University of Adelaide, Adelaide), Associate Professor Darrell Price (Pacific Private Clinic, Gold Coast), Associate Professor Christine Rodda (Sunshine Hospital, Melbourne), Professor David Simmons (Campbelltown Hospital, Sydney), Professor Richard Sinnott (eResearch, University of Melbourne), Dr Alan Sive (Townsville Hospital, Townsville), Dr Carmel Smart (John Hunter Children’s Hospital, Newcastle), Dr Monique Stone (Royal Darwin Hospital, Darwin), Associate Professor Steve Stranks (Australian Diabetes Society), Dr Elaine Tham (Women’s and Children’s Hospital, Adelaide), Dr Charles Verge (Sydney Children’s Hospital, Sydney), Professor Jerry Wales (Queensland Children’s Hospital, Brisbane), Associate Professor Glenn Ward (St. Vincent’s Hospital, Melbourne), Associate Professor Ben Wheeler (Dunedin School of Medicine, New Zealand), Dr Judy Williams (Bundaberg Diabetes Centre, Bundaberg), Dr Helen Woodhead (Australia and New Zealand Society for Paediatric Endocrinology and Diabetes), Dr Nick Woolfield (Caboolture Hospital, Caboolture), Dr Anthony Zimmermann (Lyell McEwin Hospital, Elizabeth Vale).

## References

[1] Laffel LM Kanapka LG Beck RW Bergamo K Clements MA Criego A. Effect of continuous glucose monitoring on glycemic control in adolescents and young adults with type 1 diabetes: A randomized clinical trial. Jama (2020) 323(23):2388–96. doi: 10.1001/jama.2020.6940 PMC7298603 32543683

[2] Cardona-Hernandez R Schwandt A Alkandari H Bratke H Chobot A Coles N. Glycemic outcome associated with insulin pump and glucose sensor use in children and adolescents with type 1 diabetes. Data from the international pediatric registry SWEET. Diabetes Care (2021) 44(5):1176–84. doi: 10.2337/dc20-1674 33653821

[3] Tauschmann M Hermann JM Freiberg C Papsch M Thon A Heidtmann B. Reduction in diabetic ketoacidosis and severe hypoglycemia in pediatric type 1 diabetes during the first year of continuous glucose monitoring: A multicenter analysis of 3,553 subjects from the DPV registry. Diabetes Care (2020) 43(3):e40–e2. doi: 10.2337/dc19-1358 31969340

[4] Sanderson EE Abraham MB Smith GJ Mountain JA Jones TW Davis EA. Continuous glucose monitoring improves glycemic outcomes in children with type 1 diabetes: real-world data from a population-based clinic. Diabetes Care (2021) 44(9):e171–e2. doi: 10.2337/dc21-0304 34282028

[5] Sherr JL Hermann JM Campbell F Foster NC Hofer SE Allgrove J. Use of insulin pump therapy in children and adolescents with type 1 diabetes and its impact on metabolic control: comparison of results from three large, transatlantic paediatric registries. Diabetologia (2016) 59(1):87–91. doi: 10.1007/s00125-015-3790-6 26546085

[6] Zabeen B Craig ME Virk SA Pryke A Chan AKF Cho YH. Insulin pump therapy is associated with lower rates of retinopathy and peripheral nerve abnorMality. PloS One (2016) 11(4). doi: 10.1371/journal.pone.0153033 PMC4822832 27050468

[7] Steineck I Cederholm J Eliasson B Rawshani A Eeg-Olofsson K Svensson A-M. Insulin pump therapy, multiple daily injections, and cardiovascular mortality in 18 168 people with type 1 diabetes: observational study. BMJ: Br Med J (2015) 350. doi: 10.1136/bmj.h3234 PMC4476263 26100640

[8] Virk SA Donaghue KC Wong TY Craig ME. Interventions for diabetic retinopathy in type 1 diabetes: systematic review and meta-analysis. Am J Ophthalmol (2015) 160(5):1055–64.e4. doi: 10.1016/j.ajo.2015.07.024 26210869

[9] Johnson SR Cooper MN Jones TW Davis EA. Long-term outcome of insulin pump therapy in children with type 1 diabetes assessed in a large population-based case–control study. Diabetologia (2013) 56(11):2392–400. doi: 10.1007/s00125-013-3007-9 23963323

[10] Burckhardt MA Smith GJ Cooper MN Jones TW Davis EA. Real-world outcomes of insulin pump compared to injection therapy in a population-based sample of children with type 1 diabetes. Pediatr Diabetes (2018) 19(8):1459–66. doi: 10.1111/pedi.12754 30129154

[11] Sherr JL Schoelwer M Dos Santos TJ Reddy L Biester T Galderisi A. ISPAD Clinical Practice Consensus Guidelines 2022: Diabetes technologies: Insulin delivery. Pediatr Diabetes (2022) 23(8):1406–31. doi: 10.1111/pedi.13421 36468192

[12] ElSayed NA Aleppo G Aroda VR Bannuru RR Brown FM Bruemmer D. 7. Diabetes technology: standards of care in diabetes—2023. Diabetes Care (2022) 46(Supplement_1):S111–S27. doi: 10.2337/dc23-S007 PMC9810474 36507635

[13] Graham C. Continuous glucose monitoring and global reimbursement: an update. Diabetes Technol Ther (2017) 19(S3):S60–s6. doi: 10.1089/dia.2017.0096 PMC5467100 28585871

[14] Anderson JE Gavin JR Kruger DF. Current eligibility requirements for CGM coverage are harmful, costly, and unjustified. Diabetes Technol Ther (2020) 22(3):169–73. doi: 10.1089/dia.2019.0303 PMC7047118 31596132

[15] Sumnik Z Szypowska A Iotova V Bratina N Cherubini V Forsander G. Persistent heterogeneity in diabetes technology reimbursement for children with type 1 diabetes: The SWEET perspective. Pediatr Diabetes (2019) 20(4):434–43. doi: 10.1111/pedi.12833 30773756

[16] Mönkemöller K Müller-Godeffroy E Lilienthal E Heidtmann B Becker M Feldhahn L. The association between socio-economic status and diabetes care and outcome in children with diabetes type 1 in Germany: The DIAS study (diabetes and social disparities). Pediatr Diabetes (2019) 20(5):637–44. doi: 10.1111/pedi.12847 30912245

[17] Khanolkar AR Amin R Taylor-Robinson D Viner RM Warner JT Stephenson T. Young people with Type 1 diabetes of non-white ethnicity and lower socio-economic status have poorer glycaemic control in England and Wales. Diabetes Med (2016) 33(11):1508–15. doi: 10.1111/dme.13079 26802317

[18] Addala A Auzanneau M Miller K Maier W Foster N Kapellen T. A decade of disparities in diabetes technology use and hbA(1c) in pediatric type 1 diabetes: A transatlantic comparison. Diabetes Care (2021) 44(1):133–40. doi: 10.2337/dc20-0257 PMC8162452 32938745

[19] Zuijdwijk CS Cuerden M Mahmud FH. Social determinants of health on glycemic control in pediatric type 1 diabetes. J Pediatr (2013) 162(4):730–5. doi: 10.1016/j.jpeds.2012.12.010 23360562

[20] Miller KM Beck RW Foster NC Maahs DM. HbA1c levels in type 1 diabetes from early childhood to older adults: A deeper dive into the influence of technology and socioeconomic status on hbA1c in the T1D exchange clinic registry findings. Diabetes Technol Ther (2020) 22(9):645–50. doi: 10.1089/dia.2019.0393 PMC7640747 31905008

[21] Stanley JR Clarke A Shulman R Mahmud F. Mediating effects of technology-based therapy on the relationship between socioeconomic status and glycemic management in pediatric type 1 diabetes. Diabetes Technol Ther (2023) 25(3):186–93. doi: 10.1089/dia.2022.0388 36409503

[22] Australian Government. The Australian health system (2019). Available at: https://www.health.gov.au/about-us/the-Australian-health-system (Accessed February, 2023).

[23] Johnson SR Holmes-Walker DJ Chee M Earnest A Jones TW Craig M. Universal subsidized continuous glucose monitoring funding for young people with type 1 diabetes: uptake and outcomes over 2 years, a population-based study. Diabetes Care (2022) 45(2):391–7. doi: 10.2337/dc21-1666 PMC8914416 34872983

[24] JDRF Australia. Insulin pump program (2021). Available at: https://jdrf.org.au/living-with-t1d/insulin-pump-program/ (Accessed February, 2023).

[25] Arrieta A Battelino T Scaramuzza AE Da Silva J Castañeda J Cordero TL. Comparison of MiniMed 780G system performance in users aged younger and older than 15 years: Evidence from 12 870 real-world users. Diabetes Obes Metab (2022) 24(7):1370–9. doi: 10.1111/dom.14714 PMC9545031 35403792

[26] Bassi M Patti L Silvestrini I Strati MF Ponzano M Minuto N. One-year follow-up comparison of two hybrid closed-loop systems in Italian children and adults with type 1 diabetes. Front Endocrinol (2023) 14. doi: 10.3389/fendo.2023.1099024 PMC9909036 36777356

[27] Ng SM Wright NP Yardley D Campbell F Randell T Trevelyan N. Real world use of hybrid-closed loop in children and young people with type 1 diabetes mellitus—a National Health Service pilot initiative in England. Diabetic Med (2023) 40(2):1–7. doi: 10.1111/dme.15015 36424877

[28] Clapin H Phelan H Bruns L Sinnott R Colman P Craig M. Australasian diabetes data network: building a collaborative resource. J Diabetes Sci technol (2016) 10(5):1015–26. doi: 10.1177/1932296816648983 PMC5032958 27257171

[29] National Diabetes Services Scheme. Diabetes data snapshots - Type 1 diabetes as at 31 December 2022 (2022). Available at: https://www.ndss.com.au/about-diabetes/diabetes-facts-and-figures/diabetes-data-snapshots/ (Accessed March, 2023).

[30] Australian Bureau of Statistics. (2021). Available at: https://www.abs.gov.au/statistics/detailed-methodology-information/concepts-sources-methods/socio-economic-indexes-areas-seifa-technical-paper/latest-release. (Accessed January, 2023).

[31] Gearon E Backholer K Lal A Nusselder W Peeters A. The case for action on socioeconomic differences in overweight and obesity among Australian adults: modelling the disease burden and healthcare costs. Aust N Z J Public Health (2020) 44(2):121–8. doi: 10.1111/1753-6405.12970 32190950

[32] Leggett C Giles L Anderson JJA Doogue M Couper J Pena AS. Adherence to metformin is reduced during school holidays and weekends in children with type 1 diabetes participating in a randomised controlled trial. Arch Dis Child (2019) 104(9):890–4. doi: 10.1136/archdischild-2018-316303 31079072

[33] Clapin H Smith G Vijayanand S Jones T Davis E Haynes A. Moderate and severe diabetic ketoacidosis at type 1 diabetes onset in children over two decades: A population-based study of prevalence and long-term glycemic outcomes. Pediatr Diabetes (2022) 23(4):473–9. doi: 10.1111/pedi.13327 35218122

[34] Australian Bureau of Statistics. Australian Statistical Geography Standard (ASGS): Volume 5 - Remoteness Structure (2016). Available at: https://www.abs.gov.au/AUSSTATS/abs@.nsf/Lookup/1270.0.55.005Main+Features1July%202016?OpenDocument (Accessed January, 2023).

[35] Phelan H Clapin H Bruns L Cameron FJ Cotterill AM Couper JJ. The Australasian Diabetes Data Network: first national audit of children and adolescents with type 1 diabetes. Med J Aust (2017) 206(3):121–5. doi: 10.5694/mja16.00737 28208043

[36] Haynes A Bulsara MK Bower C Codde JP Jones TW Davis EA. Independent effects of socioeconomic status and place of residence on the incidence of childhood type 1 diabetes in Western Australia. Pediatr Diabetes (2006) 7(2):94–100. doi: 10.1111/j.1399-543X.2006.00153.x 16629715

[37] Burnside MJ Williman JA Davies HM Jefferies CA Paul RG Wheeler BJ. Inequity in access to continuous glucose monitoring and health outcomes in paediatric diabetes, a case for national continuous glucose monitoring funding: A cross-sectional population study of children with type 1 diabetes in New Zealand. Lancet Reg Health West Pac (2023) 31:100644. doi: 10.1016/j.lanwpc.2022.100644 36419466 PMC9676142

[38] Lin MH Connor CG Ruedy KJ Beck RW Kollman C Buckingham B. Race, socioeconomic status, and treatment center are associated with insulin pump therapy in youth in the first year following diagnosis of type 1 diabetes. Diabetes Technol Ther (2013) 15(11):929–6. doi: 10.1089/dia.2013.0132 PMC3817890 23869706

[39] Paris CA Imperatore G Klingensmith G Petitti D Rodriguez B Anderson AM. Predictors of insulin regimens and impact on outcomes in youth with type 1 diabetes: the SEARCH for Diabetes in Youth study. J Pediatr (2009) 155(2):183–9.e1. doi: 10.1016/j.jpeds.2009.01.063 19394043

[40] Lipman Terri H Hawkes Colin P. Racial and socioeconomic disparities in pediatric type 1 diabetes: time for a paradigm shift in approach. Diabetes Care (2021) 44(1):14–6. doi: 10.2337/dci20-0048 33444165

[41] Willi SM Miller KM DiMeglio LA Klingensmith GJ Simmons JH Tamborlane WV. Racial-ethnic disparities in management and outcomes among children with type 1 diabetes. Pediatrics (2015) 135(3):424–34. doi: 10.1542/peds.2014-1774 PMC4533245 25687140

[42] Addala A Hanes S Naranjo D Maahs DM Hood KK. Provider implicit bias impacts pediatric type 1 diabetes technology recommendations in the United States: findings from the gatekeeper study. J Diabetes Sci Technol (2021) 15(5):1027–33. doi: 10.1177/19322968211006476 PMC8442183 33858206

[43] Fredette ME Zonfrillo MR Park S Quintos JB Gruppuso PA Topor LS. Self-reported insulin pump prescribing practices in pediatric type 1 diabetes. Pediatr diabetes (2021) 22(5):758–65. doi: 10.1111/pedi.13213 33855806

[44] Hershey JA Morone J Lipman TH Hawkes CP. Social determinants of health, goals and outcomes in high-risk children with type 1 diabetes. Can J Diabetes (2021) 45(5):444–50.e1. doi: 10.1016/j.jcjd.2021.02.005 33863638

[45] Hill-Briggs F Adler NE Berkowitz SA Chin MH Gary-Webb TL Navas-Acien A. Social determinants of health and diabetes: A scientific review. Diabetes Care (2020) 44(1):258–79. doi: 10.2337/dci20-0053 PMC7783927 33139407

