# Supplementary material for: Socioeconomic status and diabetes technology use in youth with type 1 diabetes: a comparison of two funding models
Source: Front Endocrinol (Lausanne). 2023 Aug 21;14:1178958. doi: 10.3389/fendo.2023.1178958 (PMC10476216; doi:10.3389/fendo.2023.1178958)
Supplement: Supplementary file 1 [file DataSheet_1.docx]

Supplementary Material

**Socioeconomic status and diabetes technology use in youth with Type 1 Diabetes: a comparison of two funding models.**

**Effect of addition of remoteness variable to models**

**Pump use (adjusted relative risk)**

|  | Excluding remoteness | Including remoteness^1^ |
| --- | --- | --- |
|  | Adjusted relative risk (95% CI) | Adjusted relative risk (95% CI) |
| IRSD Quintile | |  |
| 1 | Reference | Reference |
| 2 | 1.16 (1.04, 1.31) | 1.17 (1.04, 1.31) |
| 3 | 1.19 (1.06, 1.33) | 1.18 (1.06, 1.32) |
| 4 | 1.35 (1.21, 1.50) | 1.34 (1.20, 1.49) |
| 5 | 1.51 (1.36, 1.67) | 1.48 (1.34, 1.64) |

1 Model change χ^2^ (1, 4942) = 1.054, p = 0.304

**CGM use (adjusted relative risk)**

|  | Excluding remoteness | Including remoteness^1^ |
| --- | --- | --- |
|  | Adjusted relative risk (95% CI) | Adjusted relative risk (95% CI) |
| IRSD Quintile | |  |
| 1 | Reference | Reference |
| 2 | 1.35 (1.26, 1.44) | 1.35 (1.26, 1.45) |
| 3 | 1.28 (1.19, 1.37) | 1.26 (1.17, 1.34) |
| 4 | 1.35 (1.26, 1.45) | 1.31 (1.23, 1.41) |
| 5 | 1.34 (1.25, 1.44) | 1.28 (1.20, 1.37) |

1 Model change χ^2^ (1, 4942) = 15.0, p <0.001

**
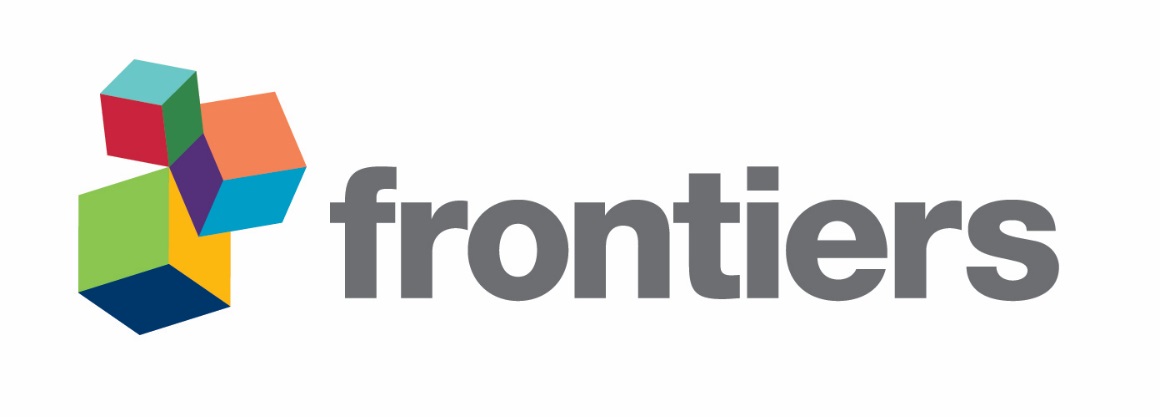
**
